# Origins of the vagal drive controlling left ventricular contractility

**DOI:** 10.1113/JP270984

**Published:** 2016-04-28

**Authors:** Asif Machhada, Nephtali Marina, Alla Korsak, Daniel J. Stuckey, Mark F. Lythgoe, Alexander V. Gourine

**Affiliations:** ^1^ Centre for Cardiovascular and Metabolic Neuroscience, Neuroscience, Physiology and PharmacologyUniversity College LondonLondonWC1E 6BTUK; ^2^ UCL Centre for Advanced Biomedical Imaging, Division of MedicineUniversity College LondonLondonWC1E 6DDUK

## Abstract

**Key points:**

- The strength, functional significance and origins of parasympathetic innervation of the left ventricle remain controversial.
- This study tested the hypothesis that parasympathetic control of left ventricular contractility is provided by vagal preganglionic neurones of the dorsal motor nucleus (DVMN).
- Under β‐adrenoceptor blockade combined with spinal cord (C1) transection (to remove sympathetic influences), systemic administration of atropine increased left ventricular contractility in rats anaesthetized with urethane, confirming the existence of a tonic inhibitory muscarinic influence on cardiac inotropy.
- Increased left ventricular contractility in anaesthetized rats was observed when DVMN neurones were silenced.
- Functional neuroanatomical mapping revealed that vagal preganglionic neurones that have an impact on left ventricular contractility are located in the caudal region of the left DVMN.
- These neurones provide functionally significant parasympathetic control of left ventricular inotropy.

**Abstract:**

The strength, functional significance and origins of direct parasympathetic innervation of the left ventricle (LV) remain controversial. In the present study we used an anaesthetized rat model to first confirm the presence of tonic inhibitory vagal influence on LV inotropy. Using genetic neuronal targeting and functional neuroanatomical mapping we tested the hypothesis that parasympathetic control of LV contractility is provided by vagal preganglionic neurones located in the dorsal motor nucleus (DVMN). It was found that under systemic β‐adrenoceptor blockade (atenolol) combined with spinal cord (C1) transection (to remove sympathetic influences), intravenous administration of atropine increases LV contractility in rats anaesthetized with urethane, but not in animals anaesthetized with pentobarbital. Increased LV contractility in rats anaesthetized with urethane was also observed when DVMN neurones targeted bilaterally to express an inhibitory *Drosophila* allatostatin receptor were silenced by application of an insect peptide allatostatin. Microinjections of glutamate and muscimol to activate or inhibit neuronal cell bodies in distinct locations along the rostro‐caudal extent of the left and right DVMN revealed that vagal preganglionic neurones, which have an impact on LV contractility, are located in the caudal region of the left DVMN. Changes in LV contractility were only observed when this subpopulation of DVMN neurones was activated or inhibited. These data confirm the existence of a tonic inhibitory muscarinic influence on LV contractility. Activity of a subpopulation of DVMN neurones provides functionally significant parasympathetic control of LV contractile function.

## Key points

- The strength, functional significance and origins of parasympathetic innervation of the left ventricle remain controversial.
- This study tested the hypothesis that parasympathetic control of left ventricular contractility is provided by vagal preganglionic neurones of the dorsal motor nucleus (DVMN).
- Under β‐adrenoceptor blockade combined with spinal cord (C1) transection (to remove sympathetic influences), systemic administration of atropine increased left ventricular contractility in rats anaesthetized with urethane, confirming the existence of a tonic inhibitory muscarinic influence on cardiac inotropy.
- Increased left ventricular contractility in anaesthetized rats was observed when DVMN neurones were silenced.
- Functional neuroanatomical mapping revealed that vagal preganglionic neurones that have an impact on left ventricular contractility are located in the caudal region of the left DVMN.
- These neurones provide functionally significant parasympathetic control of left ventricular inotropy.

**Abbreviations**
ABP: arterial blood pressure
ACh: acetylcholine
AlstR: allatostatin receptor
DVMN: dorsal vagal motor nucleus
Ees: end‐systolic elastance
eGFP: enhanced green fluorescent protein
LV: left ventricle
LVd*P*/d*t*_max_: maximum of the first differential of LVP
LVEDP: left ventricular end diastolic pressure
LVESP: left ventricular end systolic pressure
LVP: left ventricular pressure
LVV: lentiviral vector
MAP: mean arterial pressure

## Introduction

The heart is controlled by the parasympathetic and sympathetic limbs of the autonomic nervous system. Sympathetic nerves innervate the sinoatrial and atrioventricular nodes, the atria, ventricles and the conducting tissue. Parasympathetic efferent fibres are known to control nodal tissues and atria. The role of the vagal innervation of the ventricles, however, remains controversial (Coote, 2013). The majority of physiology textbooks state that the vagal innervation of the ventricle is sparse and direct parasympathetic control of ventricular contractility is insignificant. This view persists in both the scientific and the educational literature despite evidence obtained in various species (from mouse to human) demonstrating the presence of choline acetyltransferase‐positive nerve fibres, acetylcholinesterase and muscarinic receptors in both ventricles (for a recent review see Coote, 2013). Rich cholinergic innervation of epicardial and endocardial ventricular surfaces was demonstrated in human (Kent *et al*. 1974; Pauza *et al*. 2000), pig (Crick *et al*. 1999; Ulphani *et al*. 2010) and rat (Mastitskaya *et al*. 2012) hearts.

Although the functional significance of direct parasympathetic influence on ventricular contractility at resting conditions remains unknown, data obtained in dogs, pigs and humans have demonstrated that electrical stimulation of the vagus nerve decreases the force of ventricular contraction independent of heart rate changes (Eliakim *et al*. 1961; Degeest *et al*. 1965 *c*; Lewis *et al*. 2001). These data are also supported by the results of *in vitro* studies conducted on isolated rat (McMorn *et al*. 1993), cat (Hommers *et al*. 2003), guinea‐pig (Zang *et al*. 2005) and ferret (Ito *et al*. 1995; Dobrzynski *et al*. 2002) cardiomyocytes. For example, ferret ventricular cardiomyocytes respond to a prototypical effector molecule of the parasympathetic nervous system – acetylcholine (ACh) – with a reduction in twitch magnitude (Dobrzynski *et al*. 2002). These effects of ACh are mediated via rapid phosphorylation of certain inwardly rectifying potassium channels (in particular Kir3.1 and Kir3.4, expressed throughout the heart including the ventricles) (Dobrzynski *et al*. 2001), potentiation of K^+^ currents and/or reduction of Ca^2+^ currents (Dobrzynski *et al*. 2002).

Neuronal tracing studies conducted in rats (Nosaka *et al*. 1979, 1982; Stuesse, 1982; Izzo *et al*. 1993; Cheng & Powley, 2000; Sampaio *et al*. 2014), cats (Sugimoto *et al*. 1979; Ciriello & Calaresu, 1980; Geis & Wurster, 1980; Kalia & Mesulam, 1980; Bennett *et al*. 1981; Geis *et al*. 1981; Miura & Okada, 1981; Ciriello & Calaresu, 1982; Ford *et al*. 1990; Jones *et al*. 1995), dogs (Bennett *et al*. 1981; Hopkins & Armour, 1982, 1984; Plecha *et al*. 1988) and pigs (Hopkins *et al*. 1984, 1997) identified cardiac vagal preganglionic neurones within the brainstem nucleus ambiguus and the dorsal vagal motor nucleus (DVMN). Neurones of the nucleus ambiguus have rhythmic, respiratory‐related patterns of discharge with B fibre axons innervating nodal tissue and their rhythmic activity underlies the respiratory sinus arrhythmia (McAllen & Spyer, 1976; McAllen & Spyer, 1978 *a*,*b*; Ciriello & Calaresu, 1982). The relative proportion of DVMN neurones projecting to the heart is believed to be low (Jones, 2001) and their functional significance in the control of cardiac function remains unknown. DVMN neurones have slowly conducting C fibre (non‐myelinated) axons (Ciriello & Calaresu, 1980; Nosaka *et al*. 1982; Jones *et al*. 1995, 1998) and their activity is not respiratory modulated. There is evidence that these neurones protect (via a muscarinic mechanism) ventricular cardiomyocytes against acute ischaemia/reperfusion injury (Mastitskaya *et al*. 2012) and control ventricular excitability (Machhada *et al*. 2015), suggesting that the DVMN contains vagal preganglionic neurones that innervate the left ventricle (LV).

In the present study we used an anaesthetized rat model to test the hypothesis that DVMN vagal preganglionic neurones contribute to the control of left ventricular contractility. First we confirmed the presence of tonic vagal influence on left ventricular inotropy (revealed by systemic muscarinic receptor blockade). Next we determined the effect of selective silencing of DVMN neurones on left ventricular contractility and finally identified the anatomical location of the functional subpopulation of the DVMN neurones that are responsible for parasympathetic control of the LV.

## Methods

All the experiments were performed in accordance with the European Commission Directive 2010/63/EU (European Convention for the Protection of Vertebrate Animals used for Experimental and Other Scientific Purposes) and the UK Home Office (Scientific Procedures) Act (1986) with project approval from the Institutional Animal Care and Use Committee.

### Animal preparation

Adult male Sprague‐Dawley rats (380–450 g; Charles River, Oxford, UK) were anaesthetized with urethane (1.3 g kg^−1^ i.p.; following 4% isoflurane induction) or pentobarbitone sodium (induction 60 mg kg^−1^ i.p.; maintenance 10–15 mg kg^−1^ h^−1^ i.v.). As anaesthesia is known to have a significant impact on vagal activity, experiments designed to reveal tonic parasympathetic influence on the LV (Experiments 1–3) were conducted in animals anaesthetized with urethane which has been shown to preserve the level of chronotropic vagal tone similar to that in decerebrate animals (O'Leary & Jones, 2003). Experiments designed to recruit vagal activity (Experiment 3) were conducted in rats anaesthetized with pentobarbital which reduces vagal tone (O'Leary & Jones, 2003).

**Figure 1:**
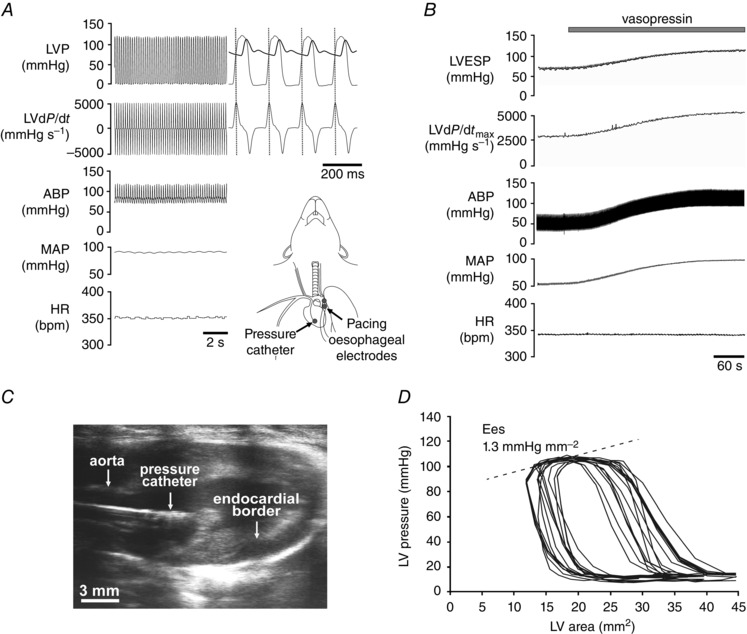
**Experimental approaches used for the assessment of left ventricular (LV) contractility in anaesthetized rats** *A*, representative recordings obtained in a pentobarbital‐anaesthetized rat illustrating simultaneous monitoring of heart rate (HR), arterial blood pressure (ABP), left ventricular pressure (LVP), derived mean arterial blood pressure (MAP) and the first differential of left ventricular pressure (LVd*P*/d*t*). *Inset*, schematic drawing illustrating placement of a 2F Millar pressure catheter in the LV chamber (advanced via the right common carotid artery) and positioning of atrial pacing electrodes in the lumen of the oesophagus at the level of the atria. *B*, representative example of simultaneous monitoring of HR, MAP, ABP, maximum of the first differential of the LV pressure (LVd*P*/d*t*_max_) and LV end‐systolic pressure (LVESP) in conditions of systemic β‐adrenoceptor blockade and spinal cord transection at C1 level before and during intravenous infusion of vasopressin (0.15 nm, 10 μl min^−1^) to restore MAP to ∼100 mmHg. Normal arterial pH, P CO 2 and $P_{O_{2}}$ were maintained by adjusting parameters of mechanical ventilation and providing supplemental oxygen in the inspired gas mixture. Sympathetic blockade and vasopressin infusion provide constant loading conditions and fixed filling times whilst pacing, allowing the use of LVd*P*/d*t*_max_ as a measure of LV contractility. *C*, a B‐mode ultrasound image of a parasternal long axis view of the LV with a Millar pressure catheter placed in the LV chamber for simultaneous recordings of LVP and LV area. *D*, representative LV pressure–area relationships obtained in a urethane‐anaesthetized rat under systemic β‐adrenoceptor blockade (atenolol). Atrial pacing at 400 bpm was combined with occlusion of the inferior vena cava to provide a family of pressure–area loops. To determine end‐systolic elastance (Ees), a regression line was drawn between the LVESP–area points of each loop ensuring *r* ^2^>0.9.

Adequate anaesthesia was ensured throughout the experiment through continuous monitoring of heart rate and arterial blood pressure (ABP) stability and the absence of a withdrawal response to a paw pinch. With an animal in a supine position, the femoral artery and both femoral veins were cannulated for measurement of systemic ABP, fluid infusion and administration of drugs. A 2F Millar pressure catheter (SPR‐320NR, Millar Instruments, Houston, TX, USA) was advanced via the right carotid artery and positioned within the chamber of the LV to monitor changes in LV pressure (LVP) (Fig. 1 *A*). The trachea was cannulated and the animal was ventilated with room air using a small rodent ventilator (Model 683, Harvard Apparatus, Holliston, MA, USA) with a tidal volume of ∼8–10 ml kg^−1^ set at a normal respiratory frequency of ∼60 strokes min^−1^. Body temperature was maintained with a servo‐controlled heating pad at 37.0 ± 0.5°C. Partial pressures of O_2_ and CO_2_ as well as pH of the arterial blood were measured every hour (348EX RAPIDLab blood gas analyser, Siemens, London, UK). The rate and volume of mechanical ventilation were adjusted and oxygen was added to the respiratory gas mixture (if required) to maintain blood gases within the physiological ranges. ABP, LVP, tracheal pressure and standard lead II ECG were recorded using a Power1401 interface and *Spike2* software (Cambridge Electronic Design, Cambridge, UK). Average waveforms were used to determine systolic blood pressure, diastolic blood pressure, mean arterial blood pressure (MAP), LV end systolic pressure (LVESP) and LV end diastolic pressure (LVEDP). The differential of LV pressure (LVd*P*/d*t*) was derived from the LVP recording using the slope function.

### Assessment of left ventricular contractility

The atria were paced at a rate of 10 or 20% above the resting heart rate using silver wire electrodes advanced via the oesophagus to the level of the heart (Fig. 1 *A*). To remove sympathetic influences, the β‐adrenoceptor blocker atenolol was administered (initial bolus dose 2 mg kg^−1^ i.v., followed by 3 mg kg^−1^ h^−1^ i.v. infusion) and the spinal cord was transected at the cervical level (C1). To restore arterial blood pressure (MAP ∼100 mmHg) after C1 transection, vasopressin (0.15 nm in saline) was infused intravenously at a rate of 10 μl min^−1^ (Fig. 1 *B*). The maximum of the first differential of LVP (LVd*P*/d*t*_max_, mmHg s^−1^) measures the inotropic state but with a caveat of load dependency. Systemic β‐adrenoceptor blockade, C1 transection and vasopressin infusion remove sympathetic influences, provide constant loading conditions and fixed filling times (whilst pacing), allowing the use of LVd*P*/d*t*_max_ as a measure of LV contractility (further assessment of the LV contractile function by indexing of the maximum of the first differential to instantaneous LVP [(LV*dP*/d*t*_max_)/LVP] or deriving the maximum instantaneous ratio of this [(LVd*P*/d*t*)/LVP]_max_ was not used due to known limitations (Van den Bos *et al*. 1973)).

### Ultrasound imaging of the left ventricle

LV pressure–area loop analysis was performed by combined measurement of LVP using a 2F Millar pressure catheter and LV imaging using a Vevo 2100 ultrasound system (VisualSonics, Amsterdam, the Nertherlands). A 24 MHz microscan transducer was used to provide a parasternal long‐axis view (Fig. 1 *C*). Precautions were taken to minimize the effect of cardiac preload using the approach previously described (Lewis *et al*. 2001). With a suture positioned sub‐diaphragmatically around the inferior vena cava as a snare to lower systolic blood pressure by ∼25 mmHg while pacing at a rate of 10% above resting heart rate, LVP–area data were acquired in segments each comprising 15 heart beats (Fig. 1 *D*). Measurements were made before and after systemic β‐adrenoceptor blockade with atenolol (3 mg kg^−1^ i.v.) and again following systemic muscarinic receptor blockade with atropine methyl nitrate (2 mg kg^−1^; i.v.). Using LV cross‐sectional area (mm^2^) as a surrogate measure of LV volume, the regression slope of the end‐systolic LVP–area relationship was used to determine the end‐systolic elastance (Ees, mmHg mm^−2^; Fig. 1 *D*) – an index of LV inotropic state which is largely insensitive to loading conditions.

### Genetic targeting of the DVMN vagal pre‐ganglionic neurones

Vagal preganglionic neurones of the DVMN characteristically express the transcriptional factor Phox2 and can be targeted to express the gene of interest using viral vectors with Phox2‐activated promoter PRSx8 (Lonergan *et al*. 2005). DVMN neurones along the whole rostro‐caudal extent of the left and right nuclei were transduced with lentiviral vectors (LVVs) to express either the G_i_‐protein‐coupled *Drosophila* allatostatin receptor (AlstR) or enhanced green fluorescent protein (eGFP, control). The plasmid pTYF‐PRSx8‐AlstR‐IRES2‐eGFP was cloned into the LVV. LVV titres were between 1×10^9^ and 1×10^10^ transducing units ml^−1^. Viral concentration and titration were carried out as described by Coleman *et al*. (2003). Validation of the transgene specificity in targeting DVMN neurones to express AlstR and efficacy of the natural ligand of AlstR, the insect peptide allatostatin, to produce a highly selective, rapid and reversible silencing of neurones expressing AlstR have been described in detail previously (Mastitskaya *et al*. 2012).

Young male Sprague‐Dawley rats (50–60 g) were anaesthetized with ketamine (60 mg kg^−1^ i.m.) and medetomidine (250 μg kg^−1^ i.m.). Adequate depth of surgical anaesthesia was confirmed by the absence of a withdrawal response to a paw pinch. With the head fixed prone in a stereotaxic frame, lignocaine (1 ml of 0.2% solution; Norbrook, Corby, UK) was injected subcutaneously before a midline dorsal neck incision was made to expose the atlanto‐occipital membrane and then the dorsal surface of the brainstem. DVMN was targeted bilaterally with two microinjections per side (0.25 μl at a rate of 0.05 μl min^−1^) of a solution containing LVV‐PRSx8‐AlstR‐IRES‐eGFP or LVV‐PRSx8‐eGFP (control). To limit potential transfection of the neighbouring A2 neurones, microinjections of viral vectors were placed ∼0.1 mm ventral to the DVMN. This limited/prevented diffusion of viral particles to the nucleus of the solitary tract while sparing hypoglossal motoneurones which do not express Phox2. Taking the calamus scriptorius as the reference point, the injections were made at 0.5 mm rostral, 0.6 mm lateral, 0.8 mm ventral and at 1.0 mm rostral, 0.8 mm lateral, 0.6 mm ventral. Anaesthesia was reversed with atipamezole (1 mg kg^−1^ i.m.). For post‐operative analgesia, rats were administered with buprenorphine (0.05 mg kg^−1^ day^−1 ^s.c.) for 3 days. Rats weighed 380–450 g at the time of the experiments.

### Glutamate and muscimol microinjections into the DVMN

The animal was anaesthetized and instrumented as described above and the head was fixed in the stereotaxic frame dorsal side up. An occipital craniotomy was performed and the cerebellum was partially removed to expose the dorsal surface of the brainstem. A three‐barrelled glass micropipette (tip size 20–25  μm) was used for randomized microinjections of either glutamate or muscimol in three discrete locations along the rostro‐caudal extent of the left and right DVMN. Taking the calamus scriptorius as the reference point, the microinjections were made at 1.3 mm rostral, 0.8 mm lateral, 0.9 mm ventral (*rostral* area), 0.8 mm rostral, 0.7 mm lateral, 0.7 mm ventral (*intermediate* area) and 0.3 mm rostral, 0.7 mm lateral, 0.7 mm ventral (*caudal* area). The barrels of the micropipette contained glutamate or muscimol, vehicle and saline containing 5% of fluorescent beads (Invitrogen, Paisley, UK). The injections were made using pressure over a period of 5–10 s and were monitored using a dissecting microscope with a calibrated micrometer disc.

### Histology

At the end of the experiments the rats were perfused through the ascending aorta with 0.9% NaCl solution followed by 500 ml 4% phosphate‐buffered (0.1 m, pH 7.4) paraformaldehyde; the brains were removed and stored in the same fixative overnight at 4°C. After cryoprotection in 30% sucrose, the brainstem was isolated and a sequence of transverse slices (30 μm) was cut. Identification of the transduced DVMN neurones was enhanced by eGFP immunostaining as described previously (Marina *et al*. 2010, 2011; Mastitskaya *et al*. 2012). Brainstem sections were first incubated with chicken anti‐GFP antibody (1:1000, Avés, Tigard, OR, USA) for 72 h followed by incubation with donkey anti‐chicken Alexa Fluor488 (1:1000, Molecular Probes, Carlsbad, CA, USA). The anatomical reconstruction of DVMN distribution of eGFP‐ and AlstR/eGFP‐expressing neurones was performed manually using the stereotaxic atlas of Paxinos & Watson (1998) as a guide. Transduced neurones were counted on both sides of the DVMN regardless of fluorescence intensity. Brainstem diagrams were assembled to illustrate the average numbers of unilaterally transduced neurones per 30 μm slice from each region of the DVMN.

### Experiment 1: to determine the effect of systemic muscarinic receptor blockade on left ventricular contractility

The animals were anaesthetized with urethane (maintains a level of vagal tone) or pentobarbital (reduces vagal tone), prepared as described above and left to stabilize for 15–30 min before measurements of baseline cardiovascular variables were taken. The effect of systemic muscarinic receptor blockade (atropine methyl nitrate, 2 mg kg^−1^ i.v.) on LV contractility was evaluated during atrial pacing (10% above resting heart rate) in conditions of systemic β‐adrenoceptor blockade (atenolol) combined with C1 transection to remove sympathetic influences (sympathetic blockade). In a separate cohort of animals, LV pressure recordings and ultrasound LV area imaging were performed followed by LV pressure–area loop analysis to determine the effect of atropine methyl nitrate on LV end‐systolic elastance.

### Experiment 2: to determine the effect of DVMN silencing on left ventricular contractility

The animals transduced to express AlstR/eGFP or eGFP by the DVMN neurones were anaesthetized with urethane and prepared as described above. To achieve acute and selective inhibition of DVMN neurones expressing AlstR, a small occipital craniotomy was performed and a miniature polyethylene catheter was placed in the cisterna magna to deliver allatostatin (4 μl of 100 μm solution in artificial cerebrospinal fluid). The animal was left to stabilize for 15–30 min. The measurements of cardiovascular variables were taken before and after allatostatin application in rats expressing AlstR/eGFP (DVMN silencing) or eGFP (control).

### Experiment 3: to identify the region of the DVMN responsible for the control of left ventricular contractility

The animals were anaesthetized with pentobarbital (reduces vagal tone) and prepared as described above. The dorsal surface of the brainstem was exposed (to aid microinjections into the DVMN) and the animal was left to stabilize for 15–30 min. Neurones in discrete locations along the rostro‐caudal extent of the left and right DVMN were activated following microinjections of an excitatory amino acid, l‐glutamate (10 mm, 40 nl, pH 7.4), and the effects of these injections on cardiovascular variables were assessed at resting heart rate conditions, during atrial pacing (10% above resting heart rate) and under systemic β‐adrenoceptor blockade (atenolol) combined with C1 transection to remove sympathetic influences. In a separate cohort of animals anaesthetized with urethane, caudal DVMN area (identified to have a significant impact on LV contractility as a result of studies with the use of glutamate microinjections) was inhibited by microinjection of a potent GABA_A_ receptor agonist, muscimol (0.1 m, 40 nl, pH 7.4), and the effect of this treatment on LV contractility was assessed. Microinjections of a vehicle (0.9% NaCl, 40 nl) were used to determine the effect of the delivery of a given volume. The order of DVMN microinjections was randomized between the left and right sites. The sites of DVMN microinjections were identified histologically and mapped using the stereotaxic atlas of Paxinos & Watson (1998).

All drugs and reagents used in this study were obtained from Sigma (Poole, UK) unless otherwise stated.

### Data analysis

Recordings of the cardiovascular variables were analysed using *Spike2* software (Cambridge Electronic Design). LV pressure–area loop analysis was performed using Lab Chart 8 software (ADInstruments, Oxford, UK). Differences between the experimental groups were assessed using GraphPad Prism 6 software (GraphPad Software Inc., La Jolla, CA, USA). Comparisons were made using two‐way ANOVA (followed by Sidak's *P* value correction for multiple comparisons) or Student's paired or unpaired *t* test, as appropriate. Data are reported as individual values and means ± SEM. Differences with *P* < 0.05 were considered to be significant.

## Results

### Experiment 1: to determine the effect of systemic muscarinic receptor blockade on left ventricular contractility

**Figure 2:**
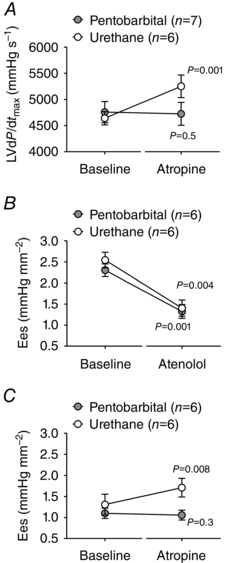
**Tonic inhibitory muscarinic influence on LV contractility is preserved under urethane anaesthesia and is abolished by pentobarbital** *A*, summary data illustrating average (means ± SEM) values of LVd*P*/d*t*_max_ obtained in conditions of atrial pacing and systemic β‐adrenoceptor blockade (atenolol) combined with C1 transection (to remove sympathetic influences) before and after intravenous administration of atropine methyl nitrate (2 mg kg^−1^) in rats anaesthetized with pentobarbital or urethane. *B*, summary data illustrating average (means ± SEM) values of end‐systolic elastance (Ees) before and after intravenous administration of atenolol (2 mg kg^−1^) in rats anaesthetized with pentobarbital or urethane. *C*, summary data illustrating average (means ± SEM) values of Ees in conditions of β‐adrenoceptor blockade (atenolol) before and after intravenous administration of atropine methyl nitrate (2 mg kg^−1^) in rats anaesthetized with pentobarbital or urethane.

In rats anaesthetized with urethane and in conditions of systemic β‐adrenoceptor blockade (atenolol) combined with C1 transection (to remove sympathetic influences), intravenous administration of atropine methyl nitrate (2 mg kg^−1^) led to significant increases in heart rate, LVd*P*/d*t*_max_, LVESP and the arterial blood pressure (Supporting Information, Table S1). Increases in LVd*P*/d*t*_max_, LVESP and MAP following atropine administration were also observed during atrial pacing (Fig. 2 *A*, Supporting Information, Table S1). In rats anaesthetized with pentobarbital, systemic atropine had no effect on cardiovascular variables during atrial pacing in conditions of sympathetic blockade (Fig. 2 *A*, Supporting Information, Table S1).

In a separate series of experiments, LV pressure–area loop analysis revealed decreases in LV contractility following systemic atenolol administration both in urethane‐anaesthetized animals (decrease in Ees from 2.5 ± 0.2 to 1.4 ± 0.2 mmHg mm^−2^; *P* = 0.004; Fig. 2 *B*) and in animals anaesthetized with pentobarbital (decrease in Ees from 2.3 ± 0.2 to 1.3 ± 0.2 mmHg mm^−2^; *P* = 0.001; Fig. 2 *B*). Subsequent systemic administration of atropine increased end‐systolic elastance in urethane‐anaesthetized (increase in Ees from 1.3 ± 0.2 to 1.7 ± 0.2 mmHg mm^−2^; *P* = 0.008; Fig. 2 *C*) but not in pentobarbital‐anaesthetized (1.1 ± 0.1 *vs*. 1.0 ± 0.1 mmHg mm^−2^; *P* = 0.3; Fig. 2 *C*) animals. These data confirm the existence of a tonic inhibitory muscarinic influence on LV contractility, which in rats is preserved under urethane anaesthesia and is suppressed by pentobarbital.

### Experiment 2: to determine the effect of DVMN silencing on left ventricular contractility

**Figure 3:**
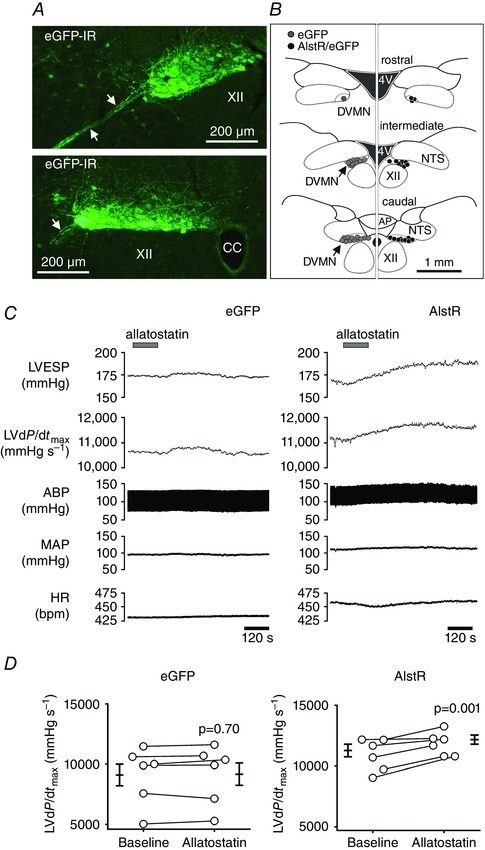
**Inhibition of vagal preganglionic neurones in the dorsal motor nucleus of the vagus nerve (DVMN) increases LV contractility** *A*, photomicrographs of the coronal sections of the rat brainstem showing expression of eGFP (amplified by immunohistochemistry) in a control animal injected with LVV‐PRSx8‐eGFP. Images illustrate representative example of the distribution of transduced DVMN neurones in the intermediate and caudal regions of the nucleus. Arrows point at the efferent DVMN fibres. XII, hypoglossal motor nucleus; eGFP‐IR, eGFP immunoreactivity; CC, central canal. *B*, averaged distribution of transduced DVMN neurones expressing eGFP (left panel) and AlstR/eGFP (right panel) 5 weeks after microinjections of LVV‐PRSx8‐AlstR‐IRES‐eGFP (*n* = 6) or LVV‐PRSx8‐eGFP (*n* = 6). Diagrams illustrate the average numbers of neurones identified to express the respective transgene in one 30 μm slice taken from the rostral (<13.3 mm caudal from Bregma), intermediate (13.3–14.0 mm caudal from Bregma) and caudal (>14.0 mm caudal from Bregma) regions of the DVMN. Each symbol represents three transduced cells. In total, 1633 ± 100 eGFP‐ and 1074 ± 79 AlstR/eGFP‐expressing neurones were identified along the rostro‐caudal extent of the left and right DVMN. No significant specific eGFP labelling was observed outside the DVMN. 4V, fourth ventricle; AP, area postrema; NTS, nucleus of the solitary tract. *C*, raw traces illustrating the recordings of HR, MAP, ABP, LVd*P*/d*t*_max_ and LVESP before and after allatostatin application into the cisterna magna of rats expressing eGFP or AlstR in the DVMN (urethane anaesthesia). No significant changes in all recorded cardiovascular variables were observed following allatostatin application in rats expressing eGFP. Inhibition of the DVMN neurones increased LVd*P*/d*t*_max_, LVESP and ABP. eGFP, animal transduced to express eGFP in the DVMN; AlstR, animal transduced to express AlstR/eGFP in the DVMN. *D*, summary data illustrating individual and average (means ± SEM) values of LVd*P*/d*t*_max_ obtained before and after allatostatin administration into the cisterna magna of rats expressing eGFP (*n* = 6) or AlstR (*n* = 6) in the DVMN (urethane anaesthesia, intact sympathetic tone).

In rats (urethane anaesthesia), bilateral inhibition of the DVMN vagal preganglionic neurones transduced to express AlstR (Fig. 3) was achieved by application of allatostatin into the cisterna magna. This experiment was conducted in animals with intact sympathetic tone. Inhibition of the DVMN neurones increased LVd*P*/d*t*_max_ (by 922 ± 232 mmHg s^−1^; *P* = 0.001; Supporting Information, Table S2), LVESP (by 16 ± 2 mmHg; *P*<0.001; Supporting Information, Table S2) and MAP (by 7 ± 4 mmHg; *P* = 0.009; Supporting Information, Table S2) (Fig. 3 *C*, *D*). DVMN inhibition had no effect on heart rate or LVEDP (Supporting Information, Table S2). No significant changes in all recorded cardiovascular variables were observed following allatostatin application into the cisterna magna in rats expressing control transgene (eGFP) in the DVMN (Supporting Information, Table S2) (Fig. 3 *C*, *D*). These data suggest that tonic activity of the DVMN vagal pre‐ganglionic neurones has a significant inhibitory effect on LV contractility.

### Experiment 3: to identify the region of the DVMN responsible for the control of left ventricular contractility

**Figure 4:**
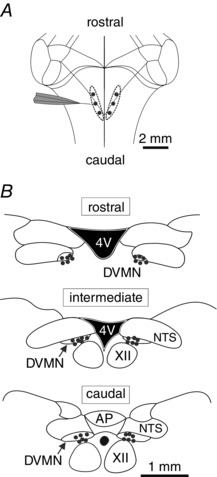
**Microinjection sites along the rostro‐caudal extent of the left and right DVMN** *A*, schematic drawing of the rat brainstem viewed from its dorsal surface illustrating targeting of the rostral, intermediate and caudal aspects of the left and right DVMN. *B*, schematic drawings of the coronal sections of the rat brainstem illustrating the locations of the rostral, intermediate and caudal microinjection sites identified by deposition of fluorescent beads. AP, area postrema; NTS, nucleus of the solitary tract; S, solitary tract.

**Figure 5:**
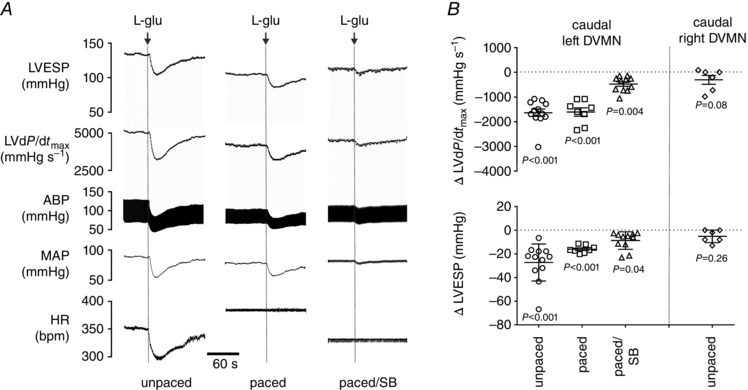
**Activation of neurones in the caudal region of the left DVMN decreases LV contractility** *A*, raw traces illustrating recordings of HR, MAP, ABP, LVd*P*/d*t*_max_ and LVESP before and after glutamate microinjections into the caudal region of the left DVMN at resting heart rate conditions, during atrial pacing and after systemic β‐adrenoceptor blockade (atenolol) combined with spinal cord (C1) transection to remove sympathetic influences (pentobarbital anaesthesia). L‐glu, l‐glutamate; SB, sympathetic blockade. *B*, summary data illustrating individual and average (means ± SEM) values of peak changes in LVESP and LVd*P*/d*t*_max_ induced by glutamate microinjections into the caudal regions of DVMN at resting heart rate conditions, during atrial pacing and during atrial pacing combined with sympathetic blockade. No significant changes in all recorded cardiovascular variables were observed following activation of the right DVMN, so the effects of glutamate microinjections were only assessed at resting conditions.

In rats anaesthetized with pentobarbital, neurones in discrete locations along the rostro‐caudal extent of the left and right DVMN (Fig. 4) were activated by glutamate microinjections and the effects of these treatments on cardiovascular variables were first assessed at resting heart rate conditions (Supporting Information, Table S3). Activation of neurones in the left caudal area of the DVMN (Fig. 4) induced decreases in LVd*P*/d*t*_max_ (by 1641 ± 134 mmHg s^−1^; *P* < 0.001), LVESP (by 28 ± 3 mmHg; *P* < 0.001), MAP (by 26 ± 3 mmHg; *P* < 0.001) and heart rate (by 20 ± 6 bpm; *P* = 0.01) (Supporting Information, Table S3; Fig. 5). Activation of the intermediate DVMN area located 0.5 mm rostrally (Fig. 4) decreased LVd*P*/d*t*_max_ (by 420 ± 112 mmHg s^−1^; *P* = 0.01; Supporting Information, Table S3) and MAP (by 10 ± 3 mmHg; *P* = 0.001; Supporting Information, Table S3), although the magnitude of the evoked changes was smaller compared to the responses elicited by glutamate actions at the caudal site. Activation of the most rostral area of the left DVMN or any area along the rostro‐caudal extent of the right DVMN had no effect on cardiovascular variables (Supporting Information, Table S3). Sites of microinjections were marked by fluorescent beads and were confirmed to lie within the targeted DVMN regions (Fig. 4 *B*).

Under constant heart rate conditions (pacing at 10% above resting heart rate) activation of neurones in the left caudal DVMN area was also associated with significant decreases in LVd*P*/d*t*_max_ (by 1536 ± 164 mmHg s^−1^; *P* < 0.001), LVESP (by 19 ± 4 mmHg; *P* < 0.001) and MAP (by 23 ± 3 mmHg; *P* < 0.001) with no effect on LVEDP (Supporting Information, Table S4, Fig. 5). Smaller, yet significant decreases in LVd*P*/d*t*_max_ (by 475 ± 140 mmHg s^−1^; *P* = 0.004), LVESP (by 9 ± 4 mmHg; *P* = 0.04) and MAP (by 8 ± 3 mmHg; *P* = 0.02) were evoked by stimulation of neurones in the left caudal DVMN under constant heart rate conditions combined with systemic β‐adrenoceptor blockade and C1 transection (Supporting Information, Table S4, Fig. 5). Responses triggered by glutamate‐induced activation of the left caudal DVMN were abolished by atropine (Supporting Information, Table S4).

**Figure 6:**
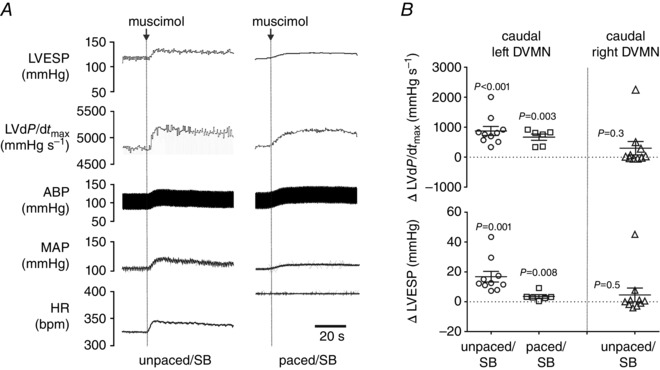
**Inhibition of neurones in the caudal region of the left DVMN increases LV contractility** *A*, raw traces illustrating recordings of HR, MAP, ABP, LVd*P*/dt_max_ and LVESP before and after muscimol microinjections into the caudal region of the left DVMN under systemic β‐adrenoceptor blockade (atenolol) combined with spinal cord (C1) transection at resting heart rate and during atrial pacing (urethane anaesthesia). *B*, summary data illustrating individual and average (means ± SEM) values of peak changes in LVESP and LVd*P*/d*t*_max_ resulting from muscimol microinjections into the caudal regions of DVMN in conditions of sympathetic blockade at resting heart rate and during atrial pacing. No significant changes in all recorded cardiovascular variables were observed following muscimol microinjections into the right caudal DVMN.

In animals anaesthetized with urethane (under systemic β‐adrenoceptor blockade combined with C1 transection), inhibition of the left caudal DVMN following microinjections of muscimol increased LVd*P*/d*t*_max_ (by 873 ± 151 mmHg s^−1^; *P* < 0.001), LVESP (by 17 ± 4 mmHg; *P* = 0.001), MAP (by 10 ± 1 mmHg; *P* = 0.001) and heart rate (by 13 ± 5 bpm; *P* = 0.004) (Supporting Information, Table S5, Fig. 6). As silencing of the DVMN neurones transduced to express AlstR by allatostatin was not associated with changes in heart rate, the cardiovascular response profile recorded after muscimol microinjections suggested potential diffusion and actions of the drug on neurones of the neighbouring brainstem structures (e.g. nucleus of the solitary tract). Smaller yet significant increases in LVd*P*/d*t*_max_ and LVESP in response to inhibition of the left caudal DVMN were observed during atrial pacing (Supporting Information, Table S5, Fig. 6). Inhibition of the right caudal DVMN by muscimol produced no changes in cardiovascular variables (Supporting Information, Table S5, Fig. 6).

## Discussion

In the present study we tested the hypothesis that tonic parasympathetic control of left ventricular contractility is provided by vagal preganglionic neurones that reside in the DVMN. First, using an anaesthetized rat model, we confirmed the presence of tonic restraining muscarinic influence on LV inotropy. Next, DVMN neurones were targeted to express inhibitory G‐protein‐coupled receptor from insects, and their selective silencing following application of the insect peptide allatostatin (Mastitskaya *et al*. 2012) was found to be associated with significant increases in LV contractility. Although vagal preganglionic neurones residing in the nucleus ambiguus are likely to have an impact on LV function, the data obtained in the present study support the hypothesis that a significant component of the parasympathetic control of LV inotropy is indeed provided by tonically active neurones of the DVMN. Functional neuroanatomical mapping, using glutamate and muscimol microinjections to activate and inhibit neuronal cell bodies in distinct locations along the rostro‐caudal extent of the left and right DVMN, identified the anatomical location of vagal preganglionic neurones that have an impact on LV contractility. These neurones appear to be concentrated in the caudal region of the left DVMN.

The strength and significance of tonic parasympathetic modulation of LV inotropic state have remained unknown. Earlier studies conducted in dogs, pigs and humans demonstrated that electrical stimulation of the vagus nerve leads to load‐independent decreases in LV contractility (Degeest *et al*. 1964, 1965 *c*; Lewis *et al*. 2001). The evidence of changes in LV inotropy observed in a reverse experimental paradigm, i.e. when acute bilateral vagotomy is performed or systemic muscarinic receptor blockade is applied, is limited. Degeest *et al*. (1965 *a*,*b*) reported that bilateral vagotomy blocks the negative inotropic effect of peripheral chemoreceptor stimulation. In humans, intracoronary administration of ACh attenuated inotropic responses to β‐adrenoceptor stimulation (dobutamine infusion) (Landzberg *et al*. 1994). Interestingly, intracoronary atropine potentiated dobutamine‐induced inotropic responses, and this effect of atropine was absent in transplanted (i.e. denervated) hearts (Landzberg *et al*. 1994). These data demonstrated the existence of a tonic restraining parasympathetic influence on LV contractility in humans. A more recent study reported increases in LVESP after acute bilateral vagotomy in the arterially perfused working heart–brainstem rat preparation (Nalivaiko *et al*. 2010). Here, using two different measures we confirmed the existence of tonic inhibitory muscarinic influence on LV contractility in a rat model. Under systemic β‐adrenoceptor blockade, intravenous administration of atropine increased LV end‐systolic elastance and LVd*P*/d*t*_max_ (at constant filling times and loading conditions). Systemic muscarinic receptor blockade increased LV contractility only in animals anaesthetized with urethane, confirming previous reports that urethane preserves, and pentobarbital reduces (chronotropic), vagal tone (O'Leary & Jones, 2003). These data illustrate a tonic inhibitory muscarinic influence on LV contractility and sensitivity of this influence to anaesthetic agents, which suppress parasympathetic tone.

In an earlier study we found that the increased activity of DVMN vagal preganglionic neurones protects LV cardiomyocytes against acute ischaemia/reperfusion injury. This cardioprotective effect was found to be mediated by a muscarinic mechanism (Mastitskaya *et al*. 2012). Based on these data, we hypothesized that the same population of DVMN neurones that exerts powerful cardioprotection may also contribute to the control of ventricular contractility. Acute bilateral inhibition of AlstR‐expressing DVMN neurones along the rostro‐caudal extent of the nuclei (by allatostatin infusion) increased LVd*P*/d*t*_max_, LVESP and ABP. This effect suggested an increase in LV contractility as no changes in LVEDP or heart rate were observed. Functional neuroanatomical mapping (using microinjections of glutamate and muscimol to activate and inhibit DVMN neuronal cell bodies, respectively) revealed that vagal preganglionic neurones responsible for parasympathetic control of LV inotropic state are concentrated in the caudal region of the left DVMN.

At resting heart rate conditions (prior to β‐adrenoceptor blockade and spinal cord transection) microinjections of glutamate into the left caudal DVMN induced marked changes in heart rate and ABP, indicative of a sympathetic withdrawal. These effects could be explained by glutamate diffusion from the site of microinjection and its actions on neurones of the neighbouring nucleus of the solitary tract, which receives inputs from all cardiorespiratory afferents (Spyer, 1994) and/or acute recruitment of the inhibitory presynaptic vagal influences which control noradrenaline release from sympathetic terminals innervating the heart. The magnitude of the parasympathetic effects on heart rate is well known to depend on the strength of the sympathetic tone, indicative of the effective functional presynaptic interactions (Levy, 1971).

Activation of neurones in the caudal region of the left DVMN led to a significant decrease in LV contractility also in conditions of systemic β‐adrenoceptor blockade and spinal cord transection, suggesting that parasympathetic control of LV inotropy originates from this region of the brainstem. Microinjections of glutamate trigger transient activation of the neuronal cell bodies in the targeted brain area and have no effect on the fibres of passage (Goodchild *et al*. 1982). This was important for the design of the current study as axons of vagal preganglionic neurones of the nucleus ambiguus form hairpin loops in the vicinity of the DVMN (Jones, 2001).

The short duration and unimodal cardiovascular response profile after microinjections of glutamate suggested that activation of neurones in the neighbouring brainstem structures is unlikely. To minimize the likelihood of this, we used one‐fifth of the glutamate concentration originally described (Goodchild *et al*. 1982) and one‐tenth of the glutamate concentration used to recruit vagal preganglionic neurones of the nucleus ambiguus (Sampaio *et al*. 2003). Interestingly, relative decreases in LV contractility triggered in response to activation of the neuronal cell bodies in the left caudal DVMN were quantitatively similar (∼20%) to those observed in rabbits when vagus nerve stimulation was combined with the anodal block technique for selective stimulation of unmyelinated efferent fibres (Garcia Perez & Jordan, 2001). These data support the conclusions of the present study, as only DVMN vagal preganglionic neurones have C fibre axons (neurones of the nucleus ambiguus have B fibre axons). Inhibition of neurones in the left caudal area of the DVMN using microinjections of a potent GABA_A_ receptor agonist, muscimol, increased LV contractility, although the magnitude of the observed effects was smaller than that induced by systemic muscarinic receptor blockade. These data suggest that either muscimol microinjections were not sufficient to inhibit the same population of DVMN neurones recruited by the actions of glutamate, or vagal preganglionic neurones residing in the nucleus ambiguus also contribute to the parasympathetic control of LV inotropy, or both.

There is evidence that cardiomyocytes themselves are able to synthesize ACh and ACh synthesis by cardiomyocytes is facilitated by muscarinic receptor activation (Kakinuma *et al*. 2009, 2012; Sun *et al*. 2013). Despite an ongoing debate on the density of vagal innervation of the LV myocardium, there is strong evidence that vagus nerve stimulation is associated with significant increases in LV ACh content (Eliakim *et al*. 1961; Akiyama *et al*. 1994; Akiyama & Yamazaki, 2001). If cardiomyocytes are capable of producing physiologically significant amounts of ACh in an autocrine/paracrine manner (sensitive to muscarinic receptor agonism), activation of an even sparse vagal efferent innervation may have a significant impact on LV function. LV cardiomyocytes are protected against ischaemia/reperfusion injury when the activity of DVMN neurones is selectively recruited using an optogenetic approach (Mastitskaya *et al*. 2012) – the data are consistent with the evidence of a potent cardioprotective effect of ACh (which is as potent as adenosine in reducing myocardial injury) reported previously (Richard *et al*. 1995; Qian *et al*. 1996; Yamaguchi *et al*. 1997; Cohen *et al*. 2001).

In summary, the data obtained in the present study confirm the existence of a tonic inhibitory muscarinic influence on LV contractility, which in the rat is preserved under urethane anaesthesia and is abolished by pentobarbital. Functionally significant parasympathetic control of LV contractile function originates from the activity of a group of vagal pre‐ganglionic neurones residing in the caudal region of the left DVMN.

## Additional information

### Competing interests

The authors have no competing interests to disclose.

### Author contributions

A.V.G. and A.M. conceived and designed the experiments. A.M., N.M., A.K. and A.V.G. collected, assembled, analysed and interpreted the data. D.J.S. and M.F.L. interpreted the data. A.M. and A.V.G. wrote the article. All authors revised the article critically for important intellectual content.

### Funding

This work was supported by the British Heart Foundation (Ref: RG/14/4/30736) and The Wellcome Trust (Ref: 095064). MB PhD funding for A.M. was provided by the Medical Research Council and The Rosetrees Trust. A.V.G. is a Wellcome Trust Senior Research Fellow. N.M. is a British Heart Foundation Intermediate Basic Science Research fellow (Ref: FS/13/5/29927).

## Supporting information


**Table S1**. The effect of systemic muscarinic receptor blockade with atropine methyl nitrate (AMN) on cardiovascular variables in conditions of β‐adrenoceptor blockade and C1 transection to remove sympathetic influences
**Table S2**. The effect of DVMN silencing (allatostatin application) on cardiovascular variables in anaesthetised (urethane) rats
**Table S3**. The effect of DVMN activation in discrete locations along the rostro‐caudal extent of the left and right nuclei (glutamate microinjections) on cardiovascular variables in anaesthetised (pentobarbital) rats
**Table S4**. The effect of neuronal activation in the left caudal DVMN (glutamate microinjections) on cardiovascular variables at resting conditions, during atrial pacing at 10% above resting heart rate, and during pacing in conditions of β‐adrenoceptor blockade combined with C1 transection (sympathetic blockade, SB) before and after systemic administration of atropine methyl nitrate (pentobarbital‐anaesthetised rats)
**Table S5**. The effect of neuronal inhibition in the caudal DVMN regions (muscimol microinjections) on cardiovascular variables in conditions of β‐adrenoceptor blockade combined with C1 transection (urethane‐anaesthetised rats)
