# Supplementary material for: Origins of the vagal drive controlling left ventricular contractility
Source: J Physiol. 2016 Apr 28;594(14):4017–30. doi: 10.1113/JP270984 (PMC4945717; doi:10.1113/JP270984)
Supplement: Supplementary file 1 — Table S1. The effect of systemic muscarinic receptor blockade with atropine methyl nitrate (AMN) on cardiovascular variables in conditions of β‐adrenoceptor blockade and C1 transection to remove sympathetic influences Table S2. The effect of DVMN silencing (allatostatin application) on cardiovascular variables in anaesthetised (urethane) rats Table S3. The effect of DVMN activation in discrete locations along the rostro‐caudal extent of the left and right nuclei (glutamate microinjections) on cardiovascular variables in anaesthetised (pentobarbital) rats Table S4. The effect of neuronal activation in the left caudal DVMN (glutamate microinjections) on cardiovascular variables at resting conditions, during atrial pacing at 10% above resting heart rate, and during pacing in conditions of β‐adrenoceptor blockade combined with C1 transection (sympathetic blockade, SB) before and after systemic administration of atropine methyl nitrate (pentobarbital‐anaesthetised rats) Table S5. The effect of neuronal inhibition in the caudal DVMN regions (muscimol microinjections) on cardiovascular variables in conditions of β‐adrenoceptor blockade combined with C1 transection (urethane‐anaesthetised rats) [file TJP-594-4017-s001.docx]

Online Supplement

**Origins of the vagal drive controlling left ventricular contractility**

*Asif Machhada, Nephtali Marina, Daniel J. Stuckey, Mark F. Lythgoe, Alexander V. Gourine (University College London, London, UK)*

**Supplemental Table 1.** The effect of systemic muscarinic receptor blockade with atropine methyl nitrate (AMN) on cardiovascular variables in conditions of β-adrenoceptor blockade and C1 transection to remove sympathetic influences

| **Anaesthesia** | **Urethane** | | **Pentobarbital** |
| --- | --- | --- | --- |
|  |  |  |  |
|  | **Unpaced** | **Paced** | **Paced** |
|  |  |  |  |
| **n** | 6 | 6 | 7 |
| **LVd*P*/d*t* max**  **(mmHg s^-1^)** |  |  |  |
| **Saline** | 5500 ± 487 | 4642 ± 127 | 4708 ± 184 |
| **AMN** | 6894 ± 664 | 5253 ± 215 | 4760 ± 203 |
| **Mean difference** | 1394 ± 224 | 611 ± 95 | 50 ± 79 |
| **p** | 0.002 | 0.001 | 0.5 |
|  |  |  |  |
| **LVESP**  **(mmHg)** |  |  |  |
| **Saline** | 120 ± 3 | 118 ± 4 | 120 ± 3 |
| **AMN** | 138 ± 5 | 132 ± 6 | 119 ± 3 |
| **Mean difference** | 18 ± 4 | 14 ± 3 | 1 ± 2 |
| **p** | 0.006 | 0.004 | 0.5 |
|  |  |  |  |
| **LVEDP**  **(mmHg)** |  |  |  |
| **Saline** | 4 ± 2 | 5 ± 1 | 6 ± 1 |
| **AMN** | 5 ± 2 | 5 ± 1 | 5 ± 1 |
| **Mean difference** | 1 ± 1 | 0 ± 1 | 0 ± 1 |
| **p** | 0.8 | 0.4 | 0.7 |
|  |  |  |  |
| **MAP**  **(mmHg)** |  |  |  |
| **Saline** | 98 ± 2 | 95 ± 2 | 103 ± 4 |
| **AMN** | 110 ± 3 | 108 ± 3 | 101 ± 3 |
| **Mean difference** | 12 ± 2 | 13 ± 3 | 2 ± 2 |
| **p** | 0.003 | 0.004 | 0.4 |
|  |  |  |  |
| **HR**  **(bpm)** |  |  |  |
| **Saline** | 353 ± 6 |  |  |
| **AMN** | 374 ± 8 |  |  |
| **Mean difference** | 21 ± 6 |  |  |
| **p** | 0.01 |  |  |

--------------------------------------------------------------------------

HR, heart rate; LVd*P*/d*t*_max_, maximum of the first differential of left ventricular pressure; LVEDP, left ventricular end diastolic pressure; LVESP, left ventricular end systolic pressure; MAP, mean arterial blood pressure.

**Supplemental Table 2.** The effect of DVMN silencing (allatostatin application) on cardiovascular variables in anaesthetised (urethane) rats

| **Transgene** | **eGFP**  **(control)** | **AlstR** |
| --- | --- | --- |
|  |  |  |
| **n** | 6 | 6 |
| **LVd*P*/d*t* max**  **(mmHg s^-1^)** |  |  |
| **Baseline** | 9099 ± 969 | 10914 ± 537 |
| **Allatostatin** | 9170 ± 991 | 11837 ± 388 |
| **Mean difference** | 71 ± 113 | 922 ± 232 |
| **p** | 0.7 | 0.001 |
|  |  |  |
| **LVESP**  **(mmHg)** |  |  |
| **Baseline** | 146 ± 10 | 156 ± 6 |
| **Allatostatin** | 149 ± 10 | 172 ± 5 |
| **Mean difference** | 4 ± 1 | 16 ± 2 |
| **p** | 0.1 | <0.0001 |
|  |  |  |
| **LVEDP**  **(mmHg)** |  |  |
| **Baseline** | 3 ± 1 | 3 ± 1 |
| **Allatostatin** | 3 ± 1 | 3 ± 1 |
| **Mean difference** | 0 ± 1 | 0 ± 0 |
| **p** | 0.7 | 0.9 |
|  |  |  |
| **MAP**  **(mmHg)** |  |  |
| **Baseline** | 91± 4 | 102 ± 3 |
| **Allatostatin** | 98± 8 | 109 ± 2 |
| **Mean difference** | 6 ± 4 | 7 ± 4 |
| **p** | 0.2 | 0.009 |
|  |  |  |
| **HR**  **(bpm)** |  |  |
| **Baseline** | 430 ± 18 | 461 ± 8 |
| **Allatostatin** | 430 ± 18 | 466 ± 10 |
| **Mean difference** | 0 ± 2 | 5 ± 4 |
| **p** | 0.9 | 0.2 |

**Supplemental Table 3.** The effect of DVMN activation in discrete locations along the rostro-caudal extent of the left and right nuclei (glutamate microinjections) on cardiovascular variables in anaesthetised (pentobarbital) rats

|  | **Left DVMN** | | | **Right DVMN** | | | |  |
| --- | --- | --- | --- | --- | --- | --- | --- | --- |
|  |  | | |  | | | | |
|  | **Rostral** | **Intermediate** | **Caudal** | **Rostral** | **Intermediate** | **Caudal** |  |  |
|  |  |  |  |  |  |  |  |  |
| **n** | 6 | 6 | 11 | 6 | 6 | 6 |  |  |
| **LVd*P*/d*t* max**  **(mmHg s^-1^)** |  |  |  |  |  |  |  |  |
| **Saline** | 4999 ± 186 | 5232 ± 222 | 5671 ± 225 | 5132 ± 191 | 5168±219 | 5190 ± 293 |  |  |
| **Glutamate** | 4845 ± 176 | 4813 ± 252 | 4030 ± 333 | 4930 ± 219 | 4987 ± 195 | 4879 ± 457 |  |  |
| **Mean difference** | 154 ± 74 | 420 ± 112 | 1641 ± 134 | 203 ± 61 | 180 ± 109 | 312 ± 179 |  |  |
| **p** | 0.6 | 0.01 | <0.0001 | 0.3 | 0.4 | 0.08 |  |  |
|  |  |  |  |  |  |  |  |  |
| **LVESP**  **(mmHg)** |  |  |  |  |  |  |  |  |
| **Saline** | 124 ± 3 | 127 ± 5 | 125 ± 2 | 125 ± 2 | 127 ± 4 | 120 ± 2 |  |  |
| **Glutamate** | 122 ± 4 | 121 ± 5 | 97 ± 5 | 122 ± 3 | 123 ± 6 | 115 ± 3 |  |  |
| **Mean difference** | 3 ± 1 | 6 ± 2 | 28 ± 3 | 2 ± 1 | 3 ± 1 | 5 ± 2 |  |  |
| **p** | 0.8 | 0.2 | <0.0001 | 0.8 | 0.5 | 0.3 |  |  |
|  |  |  |  |  |  |  |  |  |
| **LVEDP**  **(mmHg)** |  |  |  |  |  |  |  |  |
| **Saline** | 4 ± 0 | 5 ± 1 | 4 ± 1 | 5 ± 0 | 5 ± 1 | 5 ± 1 |  |  |
| **Glutamate** | 4 ± 0 | 5 ± 1 | 4 ± 1 | 4 ± 0 | 5 ± 0 | 5 ± 1 |  |  |
| **Mean difference** | 0 ± 0 | 0 ± 0 | 1 ± 0 | 1 ± 0 | 1 ± 0 | 2 ± 2 |  |  |
| **p** | >0.9 | 0.9 | 0.2 | 0.9 | 0.9 | >0.9 |  |  |
|  |  |  |  |  |  |  |  |  |
| **MAP**  **(mmHg)** |  |  |  |  |  |  |  |  |
| **Saline** | 95 ± 2 | 99 ± 5 | 98 ± 3 | 98 ± 3 | 101 ± 5 | 102 ± 3 |  |  |
| **Glutamate** | 94 ± 3 | 89 ± 7 | 72 ± 3 | 96 ± 3 | 101 ± 6 | 98 ± 2 |  |  |
| **Mean difference** | 1 ± 1 | 10 ± 3 | 26 ± 3 | 2 ± 3 | 1 ± 2 | 4 ± 3 |  |  |
| **p** | 0.9 | 0.001 | <0.0001 | 0.6 | >0.9 | 0.3 |  |  |
|  |  |  |  |  |  |  |  |  |
| **HR**  **(bpm)** |  |  |  |  |  |  |  |  |
| **Baseline** | 385 ± 14 | 396 ± 17 | 398 ± 11 | 389 ± 18 | 401 ± 15 | 392 ± 19 |  |  |
| **Glutamate** | 383 ± 14 | 396 ± 18 | 378 ± 9 | 393 ± 15 | 402 ± 14 | 393 ± 19 |  |  |
| **Mean difference** | 0 ± 1 | 1 ± 3 | 20 ± 6 | 3 ± 4 | 0 ± 2 | 0 ± 1 |  |  |
| **p** | >0.9 | >0.9 | 0.01 | 0.3 | >0.9 | >0.9 |  |  |

**Supplemental Table 4.** The effect of neuronal activation in the left caudal DVMN (glutamate microinjections) on cardiovascular variables at resting conditions, during atrial pacing at 10% above resting heart rate, and during pacing in conditions of β-adrenoceptor blockade combined with C1 transection (sympathetic blockade, SB) before and after systemic administration of atropine methyl nitrate (pentobarbital-anaesthetised rats)

|  | **Unpaced** | **Paced** | **Paced/SB** | **Paced/SB/**  **atropine** |
| --- | --- | --- | --- | --- |
|  |  |  |  |  |
| **n** | 11 | 8 | 11 | 7 |
| **LVd*P*/d*t* max**  **(mmHg s^-1^)** |  |  |  |  |
| **Saline** | 5671 ± 225 | 6043 ± 327 | 5141 ± 261 | 4759 ± 203 |
| **Glutamate** | 4030 ± 333 | 4506 ± 252 | 4667 ± 201 | 4726 ± 219 |
| **Mean Difference** | 1641 ± 134 | 1536 ± 164 | 475 ± 140 | 33 ± 175 |
| **p** | <0.0001 | <0.0001 | 0.004 | 0.9 |
|  |  |  |  |  |
| **LVESP**  **(mmHg)** |  |  |  |  |
| **Saline** | 125 ± 2 | 126 ± 4 | 126 ± 6 | 119 ± 3 |
| **Glutamate** | 97 ± 5 | 107 ± 3 | 118 ± 5 | 118 ± 3 |
| **Mean Difference** | 28 ± 3 | 19 ± 4 | 9 ± 4 | 0 ± 5 |
| **p** | <0.0001 | 0.0002 | 0.04 | 0.9 |
|  |  |  |  |  |
| **LVEDP**  **(mmHg)** |  |  |  |  |
| **Saline** | 4 ± 1 | 4 ± 1 | 4 ± 1 | 5 ± 1 |
| **Glutamate** | 4 ± 1 | 6 ± 1 | 5 ± 1 | 5 ± 1 |
| **Mean Difference** | 1 ± 0 | 2 ± 2 | 1 ± 2 | 0 ± 2 |
| **p** | 0.2 | 0.8 | 0.9 | >0.9 |
|  |  |  |  |  |
| **MAP**  **(mmHg)** |  |  |  |  |
| **Saline** | 98 ± 3 | 96 ± 6 | 108 ± 4 | 101 ± 3 |
| **Glutamate** | 72 ± 3 | 73 ± 3 | 100 ± 4 | 101 ± 3 |
| **Mean Difference** | 26 ± 3 | 23 ± 3 | 8 ± 3 | 0 ± 4 |
| **p** | <0.0001 | <0.0001 | 0.02 | >0.9 |
|  |  |  |  |  |

**Supplemental Table 5.** The effect of neuronal inhibition in the caudal DVMN regions (muscimol microinjections) on cardiovascular variables in conditions of β-adrenoceptor blockade combined with C1 transection (urethane-anaesthetised rats)

|  | **Left caudal DVMN** | | **Right caudal DVMN** |
| --- | --- | --- | --- |
|  |  |  |  |
|  | **Unpaced** | **Paced** | **Unpaced** |
|  |  |  |  |
| **n** | 10 | 6 | 10 |
| **LVd*P*/d*t* max**  **(mmHg s^-1^)** |  |  |  |
| **Saline** | 5487 ± 286 | 4445 ± 196 | 5582 ± 308 |
| **Muscimol** | 6360 ± 303 | 4686 ± 197 | 5882 ± 368 |
| **Mean Difference** | 873 ± 151 | 240 ± 44 | 299 ± 224 |
| **p** | 0.0005 | 0.003 | 0.3 |
|  |  |  |  |
| **LVESP**  **(mmHg)** |  |  |  |
| **Baseline** | 118 ± 3 | 113 ± 3 | 117 ± 4 |
| **Muscimol** | 134 ± 4 | 116 ± 3 | 121 ± 5 |
| **Mean Difference** | 17 ± 4 | 3 ± 1 | 5 ± 5 |
| **p** | 0.001 | 0.008 | 0.5 |
|  |  |  |  |
| **LVEDP**  **(mmHg)** |  |  |  |
| **Baseline** | 4 ± 0 | 7 ± 1 | 5 ± 1 |
| **Muscimol** | 4 ± 0 | 7 ± 1 | 4 ± 1 |
| **Mean Difference** | 0 ± 1 | 0 ± 1 | 0 ± 1 |
| **p** | 0.8 | 0.7 | 0.9 |
|  |  |  |  |
| **MAP**  **(mmHg)** |  |  |  |
| **Baseline** | 93 ± 1 | 95 ± 2 | 92 ± 1 |
| **Muscimol** | 103 ± 1 | 98 ± 2 | 93 ± 4 |
| **Mean Difference** | 10 ± 1 | 2 ± 1 | 1 ± 3 |
| **p** | 0.001 | 0.06 | 0.8 |
|  |  |  |  |
| **HR**  **(mmHg)** |  |  |  |
| **Baseline** | 343 ± 11 |  | 358 ± 11 |
| **Muscimol** | 356 ± 10 |  | 363 ± 11 |
| **Mean Difference** | 13 ± 5 |  | 5 ± 2 |
| **p** | 0.004 |  | 0.4 |
